# How Can Hospital Programs be Strengthened to Enhance Achievement of VISION 2020 Objectives?

**DOI:** 10.4103/0974-9233.80697

**Published:** 2011

**Authors:** Thulasiraj Ravilla, Sanil Joseph

**Affiliations:** Lions Aravind Institute of Community Ophthalmology, Aravind Eye Care System, Madurai, Tamilnadu, India

**Keywords:** Eye Care, Eye Care Management, Hospital Management, Management, VISION 2020, Program Management

## Abstract

The global initiative, “VISION 2020 – The Right to Sight” aims to eliminate avoidable blindness by year 2020. Avoidable blindness by definition are those conditions for which we already have a treatment or a surgical procedure and often a proven strategy to either prevent or cure the condition. Thus, the challenge to realize this goal would be designing the right service delivery systems specific to the local context, organizing the required resources, coordination, and implementing and monitoring these. The key “discipline” that is required to ensure successful implementation is “Management.” To be holistic, such management inputs are required both in a program as well as the hospital setting. From a program perspective, the focus will need to be on reaching the unreached, ensuring equity, creating an enabling environment, putting in place the required infrastructure, including that for developing all cadres of the eye care team, and functionally integrating eye care into the general health system and other developmental activities. From a hospital perspective, the management process should manage the internal and external ecosystems as well as all the interfaces to the hospital. It should also put in place systems for ensuring an adequate patient flow, high productivity, quality, sustainability, and accountability. Since in many countries the notion of management in health care or more specifically in eye care is at an early stage or nonexistent, a proactive effort is required to build the management capacity quickly through a structured process.

## INTRODUCTION

Blindness is beyond just a disability; it deprives affected persons of their livelihood and independence. It strips them of their self-esteem and status in the community. The irony is that the clinical knowledge exists to cure 80% of this blindness. In most cases, resources are available, yet there is little understanding on how to get an adequate number of patients, retain staff, ensure that staff are giving their best, and ensure sustainable financing or efficient service delivery. This results in underutilized resources on one hand, and increasing blindness on the other. Under the global initiative “VISION 2020 – The Right to Sight,” the aspiration is to eliminate this “avoidable blindness;” often, what undermines the efforts is lack of leadership and appropriate systems to implement the solution effectively and in a sustainable manner.

While continuing investments in infrastructure and other resources is a reflection of the commitment to eye care, there ought to be an equal concern about the low utilization of the infrastructure and other resources. The utilization level was estimated at 25% and VISION 2020 had set a target of 90%.^1^ This year marks the midway, but the achievements are not in line with the targets or milestones in various areas. There are countless examples of institutions that are grossly underutilized due to issues like doctors getting too few patients, frustrations and difficulties in managing staff.

Increasing the pool of clinically trained professionals alone will not guarantee an effective delivery of services. Managing the functions of an eye hospital is a full-time engagement that requires professionally trained managers rather than clinical personnel doing it to the extent they have the time and inclination. Clinical personnel are already scarce and have no formal training in health care management. The current situation is characterized by the lack of effective management systems and personnel with experience in health care management, making it less likely to reach the goals of VISION 2020^2^ within the timeframe. The factors which determine the success or effectiveness of an organization are not so much the quality of infrastructure or the technical competence but the leadership and management systems which put them to effective use. This is true across all organizations and activities. Ensuring smooth running of an eye hospital and enhancing the quality and quantum of eye care would be possible only through improved management processes. Hence a thorough understanding of management as it relates to eye care and its diligent practice will both solve existing problems and preempt others from occurring. In this article, the role of management both in an eye care program setting and an eye hospital setting is explored.

## DIMENSIONS IN EYE CARE MANAGEMENT

The role of management in eye care can be viewed in two different dimensions – one relating to managing “programs” and other relating to managing “eye hospitals.”

### Program perspective

From the “program” perspective, the focus of management would be on creating an enabling environment in order to reduce the prevalence of blindness by engaging all the stakeholders and aligning various sectors. Managing programs is about design and execution. The design of the program should focus on “reaching the unreached” while ensuring equity, quality, and sustainability. The execution or implementation should focus on creating an enabling environment, ensuring that all resources are available, especially the human resources, leadership, and management systems including monitoring.

### Reaching the unreached

The first step is to understand who the unreached actually are. From a socioeconomic point of view, females, the illiterate, poor, single, and the aged are reported to be the underserved categories [Table 1].^3^ From a geographic perspective, people in rural areas, difficult terrains such as hills, and places that are sparsely populated are deprived of eye care due to access-related issues. Again from the disease point of view, because of the overwhelming focus thus far on cataract, those with refractive errors, diabetic retinopathy, glaucoma, pediatric eye problems, and low vision are yet to be reached due to the lack of effective programs. This understanding and local barriers relating to access, awareness, and affordability have to be factored in while designing the service delivery initiatives.

**Table 1:** Blindness in India

Sociodemographic variables	Adjusted OR	95% CI
Female	1.4	(1.3–1.5)
Rural	1.2	(1.1–1.4)
Illiterate	3.5	(2.3–5.3)
Not working	1.8	(1.6–2.0)

Source: Current estimates of blindness in India

Cooperation rather than competition among governmental and nongovernmental partners is what is envisioned by the VISION 2020 initiative to ensure maximum use of existing resources. The World Bank-assisted Cataract Blindness Control Program of India^4^ is an example of the success that can be achieved when adequate funds and political will are combined with all the stakeholders working in synergy.

## THE WORLD BANK-ASSISTED CATARACT BLINDNESS CONTROL PROGRAM OF INDIA

The WHO-NPCB (National Programme for Control of Blindness) survey (1986–89) reported a backlog of over 22 million blind eyes and 12 million blind people in India. The survey also revealed that 80.1% of these people were blind due to cataract.

The annual incidence of cataract blindness was estimated at about 3.8 million persons and the performance then was about 1.6–1.9 million cataract operations. Consequently, the backlog was increasing with no prospect of reducing the prevailing rate from 1.49% to 0.3% which was set as the target to be achieved by year 2000. As such, the number of cataract surgeries had to be increased to at least 3–4 million annually to have a significant impact on the backlog of cataract cases.

In 1994, in an effort to reduce the backlog of cataract surgeries, the World Bank-assisted Cataract Blindness Control Program made a commitment to perform 11 million surgeries over a period of 7 years. The strategies were to build a strong public–private partnership with subsidies for cataract surgeries performed by the voluntary sector, systematically training the existing ophthalmologists to perform IOL surgeries, equipping the government hospitals for IOL surgery, ensuring regular supply of IOLs and most importantly a decentralized management process with autonomy at the district level. Through a combination of these strategies, the program exceeded the target of 11 million and performed an estimated 15.35 million cataract operations, a majority of which were performed by the private and voluntary sectors. On the quality front, the proportion of cataract surgeries with IOL implants increased steadily during the project period and now stands at over 95%. Another move toward enhancing quality was to mandate that surgeries no longer be performed in makeshift arrangements in eye camps and must be done only in hospital settings in regular operating rooms.

**Figure 1:**
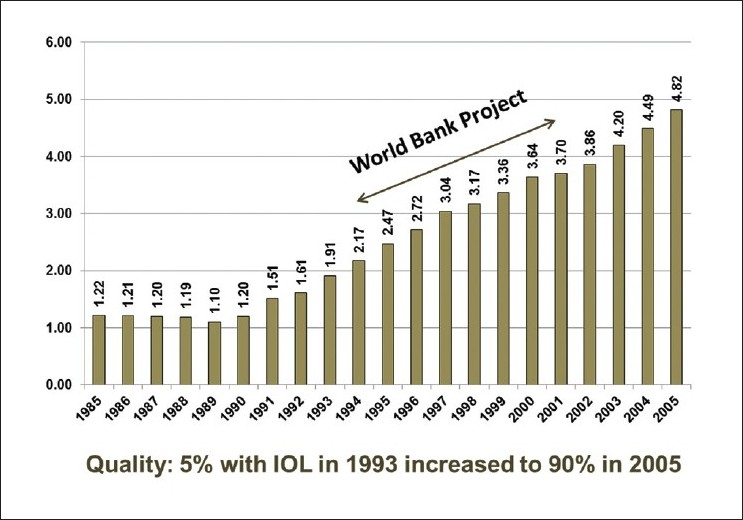
Positive outcome of stakeholders’ synergy, an example: cataract surgeries, 1985-2005

A focus on building partnerships, decentralized management, coordination, and ensuring supplies and appropriate equipment supplemented by training, monitoring, and communication systems has resulted in a program that has effectively reduced the rate of cataract blindness and cataract-related eye problems throughout India. After 8 years of program development and delivery under the World Bank assistance program, the Indian government is committed to continuing and expanding the Cataract Blindness Control Program. Cost-effectiveness had been high and a backlog of approximately 20 million cataract surgeries is under control and the country as a whole saw for the first time a decline in the overall blindness rate going down from 9.8% to 8.5% in people ≥50 years of age, and that due to cataract, reducing from 80% to 62% [Figure 1].

### Enabling environment

Researchers have reported that the impact of the ecosystems within which they operate influences the productivity of trained cataract surgeons in Eastern Africa. The variability in productivity was found to be associated with the governance of the hospital (government/mission/private), number of supporting staff, number of surgical instrument sets, means of getting supplies (donations vs. purchase), and having provision to transport patients for surgery from the outreach programs.^5^ This is a typical example of how having the right ecosystem can enhance the productivity and thereby service levels for the community in need.

Thus, creating an enabling environment is the key to making the eye care initiatives successful. The penetration of mobile phones in Africa provides an interesting example^6^ of how the holistic approach with an aim to create an enabling environment can dramatically enhance the effectiveness of a venture.

## MOBILE PHONES IN AFRICA – MARKETS AND IMPACT

**Figure 2:**
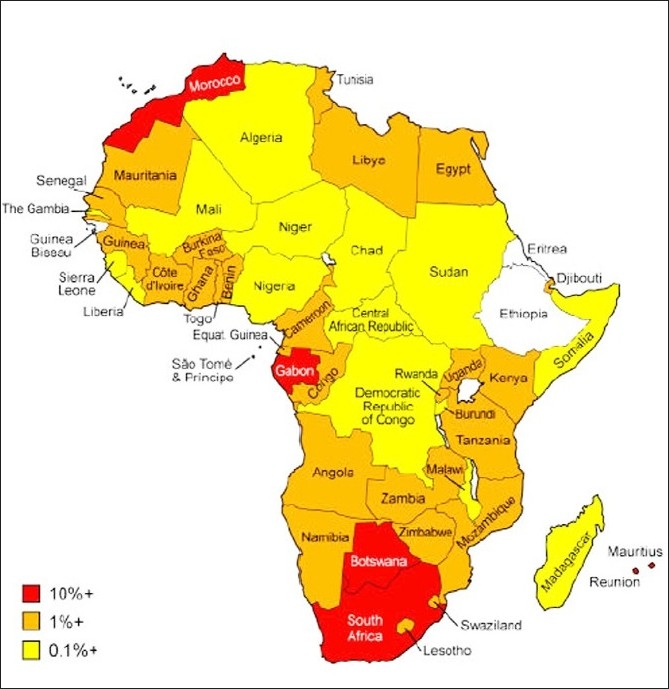
Mobile Phones Penetration in Africa

The demand for mobile phones across Africa is huge and rapidly expanding. “An Overview of Evidence” pointed out that less than 3% of the population had access to a telephone in 2001, but the number of mobile subscribers has already grown to over 50 million, representing over 7% of the population. A snapshot of the penetration of mobile phones across the continent is presented in Figure 2. The number of subscribers is currently expanding at around 35% a year, and is forecast to continue over the next few years; the trends show that current rates of growth are highest in developing countries, and the industry recognizes that its next 1 billion customers will be won by companies that develop business models that work for poorer people. This presents enormous opportunities for the delivery of propoor services.

The rapid expansion of mobile phone markets is linked to liberal regulatory environments, where operators have been given freedom to exploit demand, and competition has been effective in encouraging operators to address rural and low-income markets. Those countries with most positive e-readiness environments also have the highest mobile penetration rates [Figure 2].

The message is that the usage of mobile phones went from less than 1% in countries like Nigeria to over 40% in less than a decade with the right kind of enabling environment. Similarly, this could occur for eye care as well with the right environment.

### Human Resource Development

Human Resource Development is one of the three pillars of “VISION 2020 – The Right to Sight.” Eye care, like all medical care fields, requires a team effort. The best trained ophthalmologist still needs a team to be effective.^2^ Many countries in Africa, unlike other continents, face a severe human resource crisis due to scarcity and retention of personnel.^7^ Another problem that is common worldwide is the systems’ inability to get the best out of the individuals working in an eye care setting. This is often characterized by a critical resource person like an ophthalmologist performing a task for which he or she has no training or which can be performed just as well by persons from another category with appropriate training. All routine measurements or documentation such as refraction, ultrasound, fundus photography, visual field analysis, etc., can be performed just as well by well-trained ophthalmic technicians instead of ophthalmologists.

Similarly having trained managers permits ophthalmologists (who are not trained in management) to concentrate on doing what they do best and what they are expected to do. Ministries of Health need to recognize the critical role of management and support staff in running eye care services. They need to plan for developing such cadres of ophthalmic technicians and mangers to support the ophthalmologists who head the eye departments. This is especially critical in countries where there are a relatively few physicians who can be trained in eye care resulting in inadequate number of ophthalmologists. Without recognition of this reality and the critical role of management in delivering eye care, even hundreds of newly trained ophthalmologists will not be sufficient to achieve VISION 2020 goals in the developing world.

### Need for functional integration and a health system’s approach

Eye care has often been a standalone entity separate from the rest of health care, likely due to its limited dependence on other clinical disciplines for the diagnosis and treatment of various eye conditions. While there could be some merits in being structurally independent, there is a strong need for functional integration with other health care disciplines and with several non-health care agencies if we are to achieve the goals of VISION 2020. We need to integrate with maternal child health and the school system for effective eye care services to infants and children. Similarly, there is a need to integrate and work with physicians and specialists for treating diabetes to address the growing challenge of diabetic retinopathy. Similarly, integration with the public health department is essential to conquer onchocerciasis and trachoma.

The aim of “VISION 2020 – The Right to Sight” to eliminate avoidable blindness by year 2020 cannot be achieved by eye care services acting in isolation. Eye care staff need to engage with the wider health system, forge new alliances, identify ways to interact with their peers, influence decision makers, and advocate for the required change. A change is far more likely to occur, and be effective and sustainable if an integrated health system strengthening approach is taken.

### Hospital perspective

From the “hospital or provider” perspective, the focus needs to be on addressing the issues at the actual patient care level. This includes issues relating to generating demand, productivity and resource utilization, quality assurance, and sustainability.

### Achieving excellence – An “ecosystem” approach

**Figure 3:**
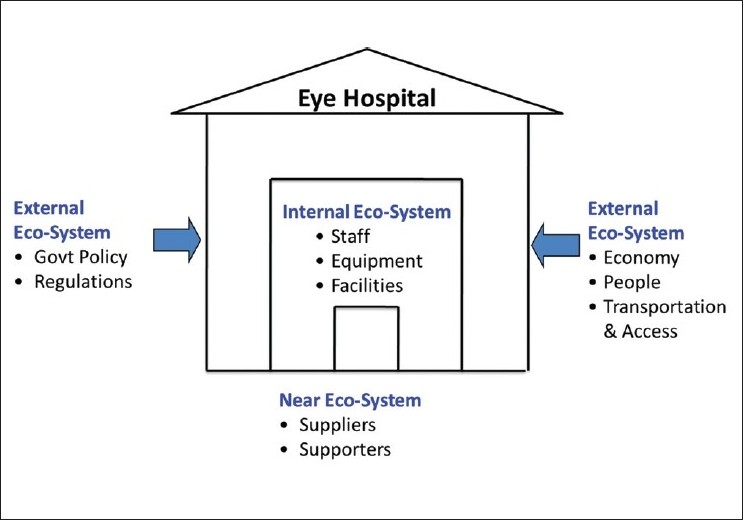
Organizational eco-systems

We need to approach the notion of hospital management in a comprehensive manner to ensure that all aspects are addressed. This is best done by categorizing the management areas as those relating to internal ecosystems, near ecosystems and external ecosystems [Figure 3].

### Internal ecosystems

This essentially relates to how one manages the various processes and resources available within the hospital at any given point of time. This will include managing the patients and their relatives who have come into the hospital, the staff present, equipment, supplies, and the infrastructure. Managing these effectively involves implementing the right workflow, processes, and monitoring systems. Since the dynamics of these change constantly, they have to be integrated with processes for continuous improvement – review performance and feedback data, identify problems and bottlenecks, brainstorm ideas, and pilot solutions, review and integrate changes or new solutions to improve work processes.

### Near ecosystems

This would include managing the providers of all the external resources such as supplies, utilities, and the other support required for day-to-day running of the hospital. How this is managed would depend on the local market conditions – depending on whether it is a highly competitive or monopolistic market. A monopolistic situation is often characterized by higher prices, uncertain delivery times and the service response is dependent on the supply organization’s culture and internal benchmarks. Managing this is very different from a situation when there is a keen competition among several suppliers for the same resource. This applies equally to the labor pool and donors.

### External ecosystems

All the hospitals are part of larger community which has its own characteristics, rules, and regulations. This would mean that the hospital will need to recognize and adapt to factors such as population density, transportation, and paying capacity on one hand and the various rules that are specific to any hospital and general laws applicable to any organization such as labour laws, building codes, etc., on the other. Additionally, the general level of peace or law-and-order situation in the service area of the hospital has to be considered as this has a direct impact both on patient access and the availability of hospital staff to work in such areas.

Ensuring that the management process addresses all three ecosystems as appropriate is critical to the smooth functioning of the hospital. Often, inadequate attention is paid to the external ecosystems in designing the services. Such organizations later assign these as the reasons for underperformance and nonachievement of goals set by them.

### Core areas in the effective management of eye care delivery

Management is a very vast field and is in continuous evolution based on the understanding and conceptualization of the principles underlying the success and failures of practices in an environment that is very dynamic. Despite this, most aspects of management can be categorized into the following broad functional domains:

1. Demand generation: In most situations, the unmet need for eye care far exceeds those who are currently seeking it. This is true regardless of whether it is a simple intervention such as dispensing spectacles for refractive errors or a more challenging condition like diabetic retinopathy. There are several mechanisms to develop the market such as internal marketing – having existing patients as ambassadors for the hospital, counseling, health education, and outreach. However, this approach fundamentally requires a developmental mindset of growing the market rather than competing for the patients already being served. In fact, the developmental approach results in a larger market share, far more effectively than reacting only on the competitive pressures. Some of the other issues relate to managing the demand in terms of smoothening seasonal variations and differentiating the services and pricing them accordingly to make them relevant and attractive to all economic strata.
2. Productivity: This relates to achieving operational excellence in a cost-effective manner. This has to do with appropriate, efficient, and optimal use of all resources which can be achieved through micro-planning, coordination, balancing of resources, lay out, patient flow, etc. The most critical and expensive resource in a hospital is the human resource and within it, the ophthalmologists. They can treat many more patients if they are not required to do tasks which can be done by others – clinical as well as administrative tasks. This not only results in higher productivity but also makes the ophthalmologists more motivated toward their work. For hospitals having cost recovery or capacity issues, productivity holds an immediate and often a no-cost solution.
3. Quality: Clinical outcomes and more importantly patient satisfaction will increasingly become the key drivers of any organization’s success, growth, and sustainability. Hence it is important to recognize that the outcome of clinical care and how it is delivered are both critical. Fundamental to ensuring quality is having appropriate measurements and putting in place processes for continuous quality improvement. In service settings such as hospitals, all these activities are carried out by the people. Hence their own wellbeing and level of satisfaction with the organization has a direct impact on the care that they deliver and its quality. Happy employees produce happy customers (patients). Hence having a well-motivated workforce is one of the key ingredients of quality. While systems, procedures, training, and such factors have a direct bearing on quality, the organizational culture and values influence how they are applied and hence quality management requires a holistic approach.
4. Sustainability: Whether the organization runs for profit or works as a non-profit it has to sustain across all dimensions such as financial, managerial, clinical services, leadership, and the community support. On the financial front, this is addressed by developing sustainable sources of income, having high efficiency, appropriate pricing, and effective cost-control measures which do not adversely affect the quality or productivity. Standard protocols, process for continuous improvements, close watch on staffing, and succession planning are methods by which the other dimensions of sustainability can be addressed.
5. Monitoring and evidence-based management: In order to ensure excellence in operations, the management team has to have an intimate knowledge of the activities and performance. This would require putting in place good monitoring and a Management Information System. The functions and activities in a hospital can be categorized as below [Table 2].

**Table 2:** Examples of functions at various levels of the organization

Levels	Example of activities
Transactional level	Registering a patient, billing, issuing supplies, etc.
Supervisory level	Resource allocation, monitoring day-to-day performance
Managerial level	Introducing new systems and procedures, hiring/ firing
Strategic level	New policies, goal/targets, new directions, etc.

**Figure 4:**
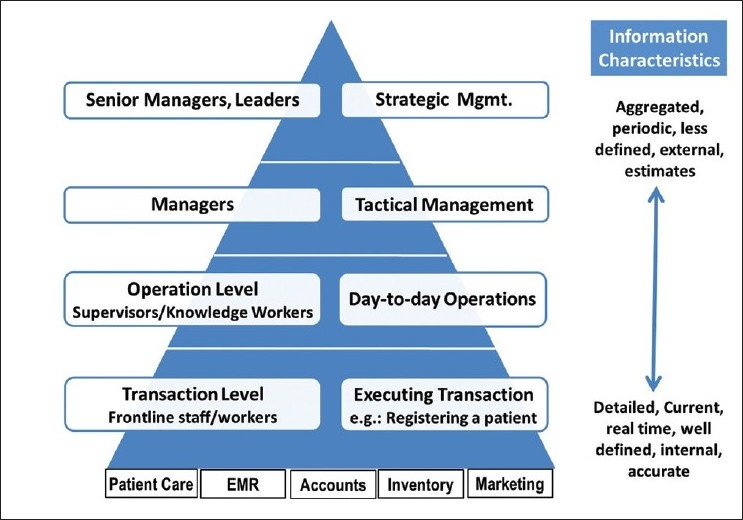
Hierarchy of information needs

The activities at all these levels are performed well and the decisions tend to be relevant when they are based on evidence which is both timely and accurate. The characteristic of the information varies depending on the level and purpose for which it is required. Thus, the monitoring process and the information systems become a critical and integral part of the whole management process [Figure 4].

In addition to having the right evidence at the right time, it is important to have a regular process by way of departmental meetings to review and reflect up on the data, make changes or improvements as needed and also closely monitor whether or not the changes actually lead to improvement.

### Building the management capacity to enhance efficiency

The global estimation puts the resource utilization of eye care facilities at 25%. All of this has now led to the realization that we need to focus on strengthening the management capacity in our eye hospitals. However, implementation remains the challenge. Even where resources are highly inadequate and the reasons for investing in human or physical infrastructure are compelling, a focus on optimal capacity utilization would lay the foundation for the proper use of new or additional resource investments.

In practical terms, the range and quantum of services to be offered by an eye hospital should reflect the needs of the community as well as the capacity in optimal conditions. The management challenge would then be to create the “optimal conditions” to achieve the targets that are set. The critical areas to focus on are fairly straightforward^6^,^7^ and would consist of ensuring:

- adequate patient volumes either through direct walk-in patients, referrals, or outreach
- efficient delivery of eye care by ensuring the right balance between resources, a high level of coordination, and right work flow and patient flow
- that both clinical outcomes and patient satisfaction are of the highest level and there is a process in place for continuous improvement
- organization’s sustainability in all dimensions – leadership, managerial, clinical, financial, and community.

In order to overcome preventable and treatable blindness, we not only need to know how to take care of our patients, but we must be able to deliver quality care through financially and otherwise sustainable systems.^8^ This can be learnt and put into practice. One of the recent initiatives of IAPB has been to raise grant funds to support a capacity building endeavor. Many international nongovernmental organizations (NGOs) have made this as one of their key strategies – eliminating avoidable blindness by enabling their partners.

## CAPACITY BUILDING PROCESS AT LIONS ARAVIND INSTITUTE FOR COMMUNITY OPHTHALMOLOGY

Lions Aravind Institute for Community Ophthalmology (LAICO) in Madurai, India, pioneered the systematic process of capacity building in other eye hospitals.^9^

The process of capacity building begins with a team from LAICO visiting a participating hospital. The team typically includes an ophthalmologist, a senior line manager, and a management specialist from LAICO, who together assess the local situation and identify opportunities for improving the organization’s infrastructure. Assessments are made in the areas of governance, management, clinical services, and outreach. In addition, the team evaluates the hospital’s system of management and organizational structure in order to identify four to six primary decision makers, who are then invited to attend the weeklong Vision and Capacity Building Workshop at Aravind Eye Hospital in Madurai, India.

During the workshop, individuals from participating hospitals are encouraged to set “stretch” goals based on how much they need to do to meet the needs of the community under ideal conditions instead of their facility’s current performance. This program presents different concepts and principles behind successful management practices as it applies to eye hospitals in each of the four areas described above.

It encourages attendees to formulate new strategies for improving the delivery of eye care which are specific and relevant to their situation. For each strategy, they must devise a plan of action, set a time frame for its completion, identify the required resources, and assign tasks to individuals. The entire process is documented online so that the team takes back the strategic plan when the members return to their hospitals. Follow-up assistance is offered through e-mail, additional site visits, and further training for individuals.

## MEASURABLE PROGRESS

Since 1994, the regional offices of many NGOs – including Lions Club International (Oak Brook, IL, USA), Sightsavers International (West Sussex, UK), Christian Blind Mission International (Bensheim, Germany), the International Eye Foundation (Kensington, MD, USA), the Seva Foundation (Berkeley, CA, USA), ORBIS International (NY, USA), and the World Health Organization (Geneva, Switzerland) – have sent representatives from their partnering eye hospitals to LAICO’s capacity building program.

Over the past decade and a half, over 270 eye hospitals have been mentored in terms of applying the principles of capacity building to develop functional systems of management. These institutions have achieved dramatic improvements within 12–18 months of completing the program by doubling their surgical output and utilizing user fees to move toward full financial self-reliance (a surrogate indicator of quality and efficiency in most circumstances).

## CONCLUSION

Health care management in general is still poorly developed in most of the developing countries. Poorly managed health systems are common, and many health workers have no formal training or exposure to organizations with good management. We are now at the mid-point of the global initiative “VISION 2020 – The Right to Sight.” Spreading awareness about the initiative, commitment of most governments, and alignment to the goal have been achieved; the human resources and infrastructure are falling in place. Now the focus must shift to management to design sustainable programs and to use these resources efficiently.^10^ It is time that resources are committed specifically for building this management capacity in eye care.
